# Trends in Racial and Ethnic Disparities in COVID-19 Hospitalizations, by Region — United States, March–December 2020

**DOI:** 10.15585/mmwr.mm7015e2

**Published:** 2021-04-16

**Authors:** Sebastian D. Romano, Anna J. Blackstock, Ethel V. Taylor, Suad El Burai Felix, Stacey Adjei, Christa-Marie Singleton, Jennifer Fuld, Beau B. Bruce, Tegan K. Boehmer

**Affiliations:** 1 CDC COVID-19 Response Team.

Persons from racial and ethnic minority groups are disproportionately affected by COVID-19, including experiencing increased risk for infection (*1*), hospitalization (*2*,*3*), and death (*4*,*5*). Using administrative discharge data, CDC assessed monthly trends in the proportion of hospitalized patients with COVID-19 among racial and ethnic groups in the United States during March–December 2020 by U.S. Census region. Cumulative and monthly age-adjusted COVID-19 proportionate hospitalization ratios (aPHRs) were calculated for racial and ethnic minority patients relative to non-Hispanic White patients. Within each of the four U.S. Census regions, the cumulative aPHR was highest for Hispanic or Latino patients (range = 2.7–3.9). Racial and ethnic disparities in COVID-19 hospitalization were largest during May–July 2020; the peak monthly aPHR among Hispanic or Latino patients was >9.0 in the West and Midwest, >6.0 in the South, and >3.0 in the Northeast. The aPHRs declined for most racial and ethnic groups during July–November 2020 but increased for some racial and ethnic groups in some regions during December. Disparities in COVID-19 hospitalization by race/ethnicity varied by region and became less pronounced over the course of the pandemic, as COVID-19 hospitalizations increased among non-Hispanic White persons. Identification of specific social determinants of health that contribute to geographic and temporal differences in racial and ethnic disparities at the local level can help guide tailored public health prevention strategies and equitable allocation of resources, including COVID-19 vaccination, to address COVID-19–related health disparities and can inform approaches to achieve greater health equity during future public health threats.

Data were obtained from the Premier Healthcare Database Special COVID-19 Release (PHD-SR),* an all-payer, administrative database containing patient-level discharge records (including discharges ending in death) from more than 800 nongovernmental, community, and teaching hospitals across the United States. The database represents 20% of U.S. hospital admissions. Analyses were limited to 655 facilities that submitted data during March–December 2020 and did not have unusual race or ethnicity reporting patterns.^†^ COVID-19 hospitalizations were defined as having *International Classification of Diseases, Tenth Revision, Clinical Modification* (ICD-10-CM) discharge diagnosis code B97.29 (other coronavirus as the cause of disease classified elsewhere [recommended before the April 1, 2020 release of U07.1]) during March–April 2020 or code U07.1 (COVID-19, virus identified) during April–December 2020. Patient race and ethnicity variables were categorized as Hispanic or Latino of any race (Hispanic), non-Hispanic Asian (Asian), non-Hispanic Black (Black), non-Hispanic White (White), non-Hispanic all other races (other race),^§^ or race or ethnicity missing (unknown). Patients with unknown race/ethnicity were not included in the trend analyses.

The cumulative proportion (percentage) of hospitalized patients with COVID-19 was calculated as the number of patients with an index COVID-19 hospitalization^¶^ during March–December 2020 divided by the total number of patients hospitalized during the same period for any reason, including COVID-19. Monthly proportions of hospitalized patients with COVID-19 were calculated as the number of patients with an index COVID-19 hospitalization during a given month divided by the number of patients with a first hospitalization for any reason during the same month. Proportions were stratified by patient race/ethnicity and by four U.S. Census regions** based on facility location. For each region, aPHRs were calculated for each racial and ethnic minority group compared with White patients using multivariable Poisson regression. Confidence intervals were calculated for the cumulative aPHRs using generalized estimating equations to account for clustering within facilities. Changes in the monthly aPHRs for each racial/ethnic group were examined qualitatively. The racial/ethnic distribution among all patients hospitalized in 2019 was compared with that among all non-COVID-19 patients hospitalized in 2020 to assess consistency of racial/ethnic proportions across pandemic and non-pandemic years. Analyses were conducted using SAS (version 9.4; SAS Institute) and R (version 4.0.2; The R Foundation). This activity was reviewed by CDC and was conducted consistent with applicable federal law and CDC policy.^††^

During March–December 2020, PHD-SR identified 3,780,251 total unique hospitalized patients, including 298,066 (7.9%) unique patients with a COVID-19 diagnosis. The racial/ethnic distributions of non-COVID-19 patient populations were similar in 2019 and 2020 (Supplementary Table https://stacks.cdc.gov/view/cdc/104959). The racial/ethnic distribution of hospitalized COVID-19 patients differed among U.S. Census regions (Table). In every region, Hispanic patients represented the highest cumulative proportion of hospitalized patients with COVID-19 and highest cumulative aPHR relative to White patients. The monthly patterns in proportions of hospitalized patients with COVID-19 by race and ethnicity varied by U.S. Census region early in the pandemic, but all regions showed increasing proportions of patients hospitalized among all racial/ethnic groups later in 2020 (Figure 1). In the Northeast, the proportion peaked in April and was high for all racial and ethnic minority groups. In the Midwest, the proportion was high among several racial and ethnic minority groups during April–May and peaked in November for all groups. In the South, the proportion among Hispanic and Black patients peaked in July. In the West, the proportion among Hispanic patients was high in July and increased more than that in other racial/ethnic groups during November–December, peaking in December.

**TABLE:** Racial/ethnic* distribution of COVID-19 and all hospitalized patients, proportion of hospitalized patients with COVID-19, and cumulative unadjusted^†^ and adjusted^§^ proportionate hospitalization ratios, by U.S. Census region^¶^ — United States, March–December 2020**

**Census region race/ethnicity**	**No. (%)**		**Percentage of hospitalized patients with COVID-19**	**Cumulative proportionate hospitalization ratios**	
	**COVID-19 hospitalized patients**	**All hospitalized patients**		**Unadjusted**	**Adjusted**
**Total**	**298,066**	**3,780,251**	**7.88**	**—**	**—**
**Northeast**					
White	20,595 (40.4)	299,166 (53.3)	6.88	Referent	Referent
Hispanic	10,589 (20.8)	75,625 (13.5)	14.00	2.0	2.7 (2.4–3.0)
Asian	1,912 (3.8)	18,406 (3.3)	10.39	1.5	2.0 (1.8–2.3)
Black	9,158 (18.0)	72,242 (12.9)	12.68	1.8	2.0 (1.9–2.2)
Other	2,327 (4.6)	19,925 (3.6)	11.68	1.7	2.1 (1.9–2.4)
Unknown	6,363 (12.5)	75,407 (13.4)	8.44	1.2	1.6 (1.5–1.9)
**Northeast total**	**50,944 (100.0)**	**560,771 (100.0)**	**9.08**	**—**	**—**
**Midwest**					
White	49,017 (65.8)	655,542 (70.0)	7.48	Referent	Referent
Hispanic	6,072 (8.2)	47,733 (5.1)	12.72	1.7	2.7 (2.5–2.9)
Asian	1,413 (1.9)	14,622 (1.6)	9.66	1.3	2.1 (1.8–2.4)
Black	12,110 (16.3)	119,165 (12.7)	10.16	1.4	1.7 (1.6–1.8)
Other	2,588 (3.5)	36,360 (3.9)	7.12	1.0	1.2 (0.9–1.7)
Unknown	3,302 (4.4)	62,543 (6.7)	5.28	0.7	1.2 (1.1–1.4)
**Midwest total**	**74,502 (100.0)**	**935,965 (100.0)**	**7.96**	**—**	**—**
**South**					
White	60,797 (42.1)	971,381 (53.2)	6.26	Referent	Referent
Hispanic	36,311 (25.1)	283,835 (15.6)	12.79	2.0	2.8 (2.5–3.3)
Asian	1,903 (1.3)	28,102 (1.5)	6.77	1.1	1.6 (1.5–1.8)
Black	31,159 (21.6)	307,445 (16.8)	10.13	1.6	1.9 (1.8–2.0)
Other	3,569 (2.5)	47,421 (2.6)	7.53	1.2	1.7 (1.5–1.9)
Unknown	10,694 (7.4)	186,962 (10.2)	5.72	0.9	1.3 (1.1–1.5)
**South total**	**144,433 (100.0)**	**1,825,146 (100.0)**	**7.91**	**—**	**—**
**West**					
White	9,056 (32.1)	207,766 (45.3)	4.36	Referent	Referent
Hispanic	10,478 (37.2)	90,759 (19.8)	11.54	2.6	3.9 (3.2–4.8)
Asian	2,029 (7.2)	34,344 (7.5)	5.91	1.4	1.5 (1.2–1.9)
Black	1,703 (6.0)	25,301 (5.5)	6.73	1.5	1.8 (1.5–2.2)
Other	2,331 (8.3)	34,899 (7.6)	6.68	1.5	2.0 (1.6–2.5)
Unknown	2,590 (9.2)	65,300 (14.2)	3.97	0.9	1.4 (1.0–1.9)
**West total**	**28,187 (100.0)**	**458,369 (100.0)**	**6.15**	**—**	**—**

**Abbreviation:** CI = confidence intervals.

* Hispanic persons could be of any race; Asian, Black, White, and Other race persons were non-Hispanic. Other group includes persons who were a race other than Asian, Black, or White, including American Indian/Alaska Native, Native Hawaiian/Pacific Islander, and multiple races. Race and ethnicity were categorized as “unknown” if race or ethnicity was missing.

^†^ The unadjusted proportionate hospitalization ratio is calculated as the percentage of hospitalized patients with COVID-19 among a racial/ethnic minority group divided by the percentage among White patients for that given region.

^§^ Adjusted for age group using Poisson regression. Age groups were: <18, 18–39, 40–54, 55–64, 65–74, and ≥75 years.

^¶^ *Northeast*: Connecticut, Maine, Massachusetts, New Hampshire, New Jersey, New York, Pennsylvania, Rhode Island, and Vermont; *Midwest*: Illinois, Indiana, Iowa, Kansas, Michigan, Minnesota, Missouri, Nebraska, North Dakota, Ohio, South Dakota, and Wisconsin; *South*: Alabama, Arkansas, Delaware, District of Columbia, Florida, Georgia, Kentucky, Louisiana, Maryland, Mississippi, North Carolina, Oklahoma, South Carolina, Tennessee, Texas, Virginia, and West Virginia; *West*: Alaska, Arizona, California, Colorado, Hawaii, Idaho, Montana, Nevada, New Mexico, Oregon, Utah, Washington, and Wyoming.

** Data from subset of 655 hospitals in Premier Healthcare Database Special COVID-19 Release.

**FIGURE 1:**
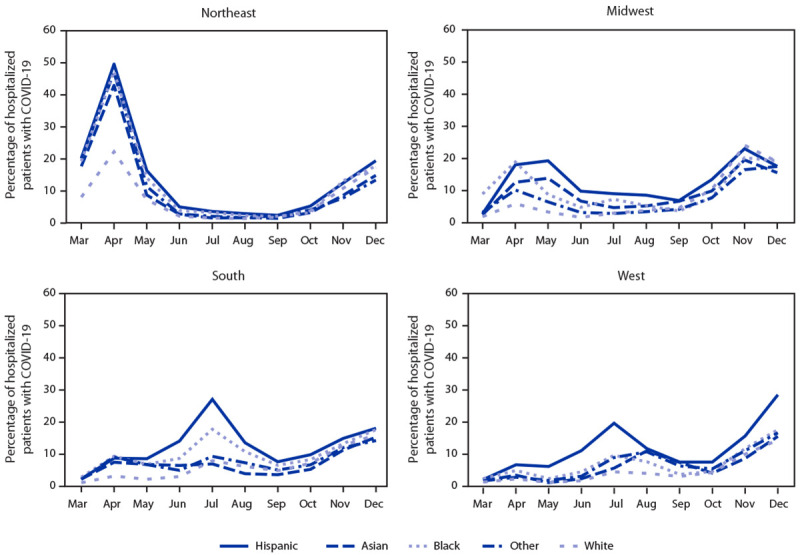
Monthly proportion (percentage) of hospitalized patients with COVID-19, by race/ethnicity* and U.S. Census region^†^ — United States, March–December 2020^§^ * Hispanic persons could be of any race; Asian, Black, White, and Other race persons were non-Hispanic. Other group includes persons who were a race other than Asian, Black, or White, including American Indian/Alaska Native, Native Hawaiian/Pacific Islander, and multiple races. ^†^ *Northeast*: Connecticut, Maine, Massachusetts, New Hampshire, New Jersey, New York, Pennsylvania, Rhode Island, and Vermont; *Midwest*: Illinois, Indiana, Iowa, Kansas, Michigan, Minnesota, Missouri, Nebraska, North Dakota, Ohio, South Dakota, and Wisconsin; *South*: Alabama, Arkansas, Delaware, District of Columbia, Florida, Georgia, Kentucky, Louisiana, Maryland, Mississippi, North Carolina, Oklahoma, South Carolina, Tennessee, Texas, Virginia, and West Virginia; *West*: Alaska, Arizona, California, Colorado, Hawaii, Idaho, Montana, Nevada, New Mexico, Oregon, Utah, Washington, and Wyoming. ^§^ Data from subset of 655 hospitals in Premier Healthcare Database Special COVID-19 Release.

Racial and ethnic disparities in the proportion of hospitalized patients with COVID-19, as measured by aPHRs, were most pronounced early in the pandemic (Figure 2). In the Northeast, relative to White patients, aPHRs were highest for most racial and ethnic minority groups in April, and remained high for Hispanic patients through July, followed by a decrease among all racial and ethnic minority groups through December. In the Midwest, relative to White patients, the aPHR for Black patients was highest in March, and the aPHRs for Asian and Hispanic patients were highest during May–June; aPHRs decreased through November, with slight increases for Black patients and patients of other race in December. In the South, aPHRs for all racial and ethnic minority groups were highest during May–June and decreased through November, except for an increase among Asian patients during September–December. In the West, aPHRs were highest for Hispanic and Black patients in June and for Asian patients and patients of other races in August; aPHRs decreased through November, with slight increases among Hispanic and Asian patients in December.

**FIGURE 2:**
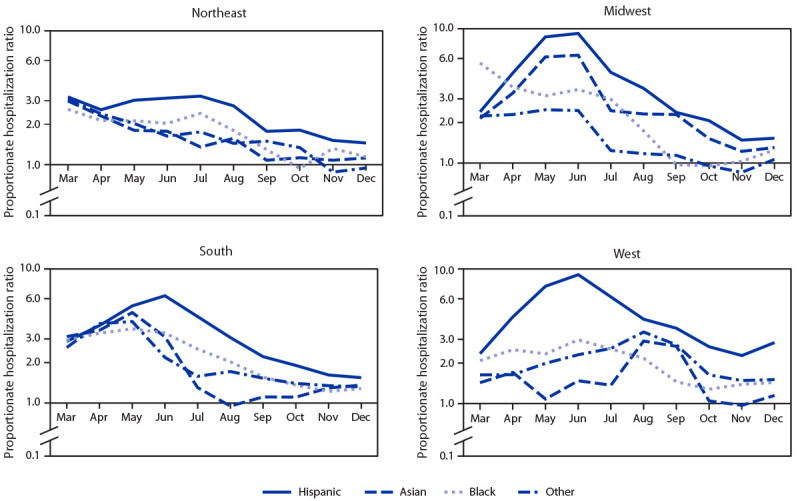
Monthly age-adjusted* COVID-19 proportionate hospitalization ratios comparing racial and ethnic minority patients^†^ with White patients, by U.S. Census region^§^ ― United States, March–December 2020^¶^ * Adjusted for age group using Poisson regression. Age groups were: <18, 18–39, 40–54, 55–64, 65–74, and ≥75 years. ^†^ Hispanic persons could be of any race; Asian, Black, White, and Other race persons were non-Hispanic. Other group includes persons who were a race other than Asian, Black, or White, including American Indian/Alaska Native, Native Hawaiian/Pacific Islander, and multiple races. ^§^ *Northeast*: Connecticut, Maine, Massachusetts, New Hampshire, New Jersey, New York, Pennsylvania, Rhode Island, and Vermont; *Midwest*: Illinois, Indiana, Iowa, Kansas, Michigan, Minnesota, Missouri, Nebraska, North Dakota, Ohio, South Dakota, and Wisconsin; *South*: Alabama, Arkansas, Delaware, District of Columbia, Florida, Georgia, Kentucky, Louisiana, Maryland, Mississippi, North Carolina, Oklahoma, South Carolina, Tennessee, Texas, Virginia, and West Virginia; *West*: Alaska, Arizona, California, Colorado, Hawaii, Idaho, Montana, Nevada, New Mexico, Oregon, Utah, Washington, and Wyoming. ^¶^ Data from subset of 655 hospitals in Premier Healthcare Database Special COVID-19 Release.

## Discussion

Analysis of hospitalizations from a database including more than 3.7 million hospital discharges and approximately 300,000 hospitalized COVID-19 patients during March–December 2020 found that racial and ethnic minority groups experienced higher proportions of COVID-19–related hospitalization compared with White patients. This finding is consistent with previous studies documenting racial and ethnic disparities in COVID-19 hospitalization (*2*,*3*) and expands upon earlier studies by documenting how these disparities have shifted over time and how they have differed by region. The largest disparities in the proportion of patients hospitalized with COVID-19 occurred early in the pandemic (April–July 2020) and became less pronounced over time as COVID-19 hospitalizations increased among White patients. However, as of December 2020, disparities remained among racial/ethnic minority groups in all regions, most notably among Hispanic patients in the West.

Racial and ethnic disparities in COVID-19 hospitalization are driven by both a higher risk for exposure to SARS-CoV-2 and a higher risk for severe COVID-19 disease (e.g., due to higher prevalence of underlying medical conditions) among racial and ethnic minority groups, both of which are influenced by social determinants of health.^§§^ The regional and temporal patterns in disparities observed in this analysis are likely driven primarily by differences between racial and ethnic minority groups and White persons in SARS-CoV-2 exposure risk associated with occupational and housing conditions and socioeconomic status (*6*,*7*). The declining racial and ethnic disparities observed in late 2020 do not necessarily reflect reduced risk for infection or improved outcomes for certain racial and ethnic minority groups, but rather an increased risk for infection and subsequent hospitalization among White patients as COVID-19 spread throughout the United States (*8*). COVID-19–related hospitalization is one of several measures that can provide insight into the impact of COVID-19 on racial and ethnic minority populations and should be interpreted in the context of other measures such as COVID-19 incidence and mortality rates. It is important to continue to monitor racial and ethnic disparities in COVID-19 infection and outcomes at national, regional, and local levels.

Changes in the provision of health care services, such as a reduction in elective procedures, during the COVID-19 pandemic could have affected the racial/ethnic distribution of hospitalized patients in 2020, which was used as the denominator for this analysis. However, a supplementary analysis found similar racial and ethnic distributions among persons hospitalized in 2019 and for hospitalizations in 2020 that were not related to COVID-19, indicating that observed disparities in 2020 COVID-19 hospitalizations were not likely due to changes in the patient population served.

The findings in this report are subject to at least three limitations. First, the underlying catchment areas for the facilities in this analysis are not known; therefore, population-based rates could not be calculated. Second, American Indian or Alaska Native patients, Native Hawaiian or Pacific Islander patients, and patients reporting multiple races were aggregated within PHD-SR into a non-Hispanic other race category to protect patient privacy. Therefore, proportions of COVID-19–related hospitalizations among these groups could not be assessed; current data show a high risk for COVID-19 infection, hospitalization, and death among American Indian or Alaska Native persons compared with White persons.^¶¶^ Finally, the study did not adjust for underlying medical conditions that increase the risk for severe COVID-19 outcomes such as hospitalization and might be more common among racial and ethnic minority groups.***

Disparities in COVID-19 hospitalization by race and ethnicity varied by U.S. Census region and became less pronounced over the course of the pandemic as the proportion of White patients hospitalized with COVID-19 increased. Identification of the specific social determinants of health (e.g., access to health care, occupation and job conditions, housing instability, and transportation challenges) that contribute to geographic and temporal differences in racial and ethnic disparities in COVID-19 infection and poor health outcomes is critical (*6*,*7*,*9*,*10*). A better understanding of these factors at a local level can help guide tailored public health prevention strategies and equitable allocation of resources, including COVID-19 vaccination, to better address COVID-19–related health disparities and can inform approaches to achieve greater health equity during future public health threats.

**Summary**

**What is already known about this topic?**

COVID-19 disproportionately affects racial and ethnic minority groups in the United States.

**What is added by this report?**

Within each U.S. Census region, the proportion of hospitalized patients with COVID-19 was highest for Hispanic or Latino patients. Racial and ethnic disparities were largest during May–July 2020 and became less pronounced as the pandemic spread throughout the country; however, disparities remained in December 2020 in all regions.

**What are the implications for public health practice?**

Understanding the social determinants of health contributing to geographic and temporal differences in racial and ethnic disparities at a local level can help guide public health prevention strategies and equitable resource allocation, including COVID-19 vaccination, to address COVID-19–related health disparities.

## Footnotes

* Data in PHD-SR, formerly known as the PHD COVID-19 Database, are released every 2 weeks; release date March 2, 2021; access date March 3, 2021. http://offers.premierinc.com/rs/381-NBB-525/images/PHD_COVID-19_White_Paper.pdf

† Facilities were excluded if they reported only one category of race (e.g., all unknown or all White) for all hospitalized patients or only one category of ethnicity (e.g., all unknown) for all hospitalized patients during the analytic period of March–December 2020 (n = 99).

§ The other races group includes persons who reported non-Hispanic ethnicity and a race other than Asian, Black, or White, including American Indian/Alaska Native, Native Hawaiian/Pacific Islander, and multiple races.

¶ Index COVID-19 hospitalization was defined a patient’s first hospitalization with a discharge diagnosis of COVID-19 (code B97.29 during March–April or U07.1 during April–December) within the March–December study period.

** *Northeast*: Connecticut, Maine, Massachusetts, New Hampshire, New Jersey, New York, Pennsylvania, Rhode Island, and Vermont; *Midwest*: Illinois, Indiana, Iowa, Kansas, Michigan, Minnesota, Missouri, Nebraska, North Dakota, Ohio, South Dakota, and Wisconsin; *South*: Alabama, Arkansas, Delaware, District of Columbia, Florida, Georgia, Kentucky, Louisiana, Maryland, Mississippi, North Carolina, Oklahoma, South Carolina, Tennessee, Texas, Virginia, and West Virginia; *West*: Alaska, Arizona, California, Colorado, Hawaii, Idaho, Montana, Nevada, New Mexico, Oregon, Utah, Washington, and Wyoming.

†† 45 C.F.R. part 46, 21 C.F.R. part 56; 42 U.S.C. Sect. 241(d); 5 U.S.C. Sect. 552a; 44 U.S.C. Sect. 3501 et seq.

§§
https://www.cdc.gov/coronavirus/2019-ncov/community/health-equity/racial-ethnic-disparities/index.html

¶¶
https://www.cdc.gov/coronavirus/2019-ncov/covid-data/investigations-discovery/hospitalization-death-by-race-ethnicity.html

***
https://www.cdc.gov/coronavirus/2019-ncov/community/health-equity/racial-ethnic-disparities/disparities-illness.html
